# Targeting Autophagy for Overcoming Resistance to Anti-EGFR Treatments

**DOI:** 10.3390/cancers11091374

**Published:** 2019-09-16

**Authors:** Yoojung Kwon, Misun Kim, Hyun Suk Jung, Youngmi Kim, Dooil Jeoung

**Affiliations:** 1 Department of Biochemistry, College of Natural Sciences, Kangwon National University, Chunchon 24341, Korea; 2 Institute of New Frontier Research, College of Medicine, Hallym University, Chunchon 24251, Korea

**Keywords:** anti-EGFR treatments, autophagy, EGFR signaling, co-targeting

## Abstract

Epidermal growth factor receptor (EGFR) plays critical roles in cell proliferation, tumorigenesis, and anti-cancer drug resistance. Overexpression and somatic mutations of EGFR result in enhanced cancer cell survival. Therefore, EGFR can be a target for the development of anti-cancer therapy. Patients with cancers, including non-small cell lung cancers (NSCLC), have been shown to response to EGFR-tyrosine kinase inhibitors (EGFR-TKIs) and anti-EGFR antibodies. However, resistance to these anti-EGFR treatments has developed. Autophagy has emerged as a potential mechanism involved in the acquired resistance to anti-EGFR treatments. Anti-EGFR treatments can induce autophagy and result in resistance to anti-EGFR treatments. Autophagy is a programmed catabolic process stimulated by various stimuli. It promotes cellular survival under these stress conditions. Under normal conditions, EGFR-activated phosphoinositide 3-kinase (PI3K)/AKT serine/threonine kinase (AKT)/mammalian target of rapamycin (mTOR) signaling inhibits autophagy while EGFR/rat sarcoma viral oncogene homolog (RAS)/mitogen-activated protein kinase kinase (MEK)/mitogen-activated protein kinase (MAPK) signaling promotes autophagy. Thus, targeting autophagy may overcome resistance to anti-EGFR treatments. Inhibitors targeting autophagy and EGFR signaling have been under development. In this review, we discuss crosstalk between EGFR signaling and autophagy. We also assess whether autophagy inhibition, along with anti-EGFR treatments, might represent a promising approach to overcome resistance to anti-EGFR treatments in various cancers. In addition, we discuss new developments concerning anti-autophagy therapeutics for overcoming resistance to anti-EGFR treatments in various cancers.

## 1. Introduction

Constitutive signaling from the EGFR promotes cell survival, proliferation [1], and invasiveness [2]. Aberrant EGFR signaling has been found in many human malignancies, including colorectal, lung, breast, and head and neck cancer [3,4]. Overexpression and activating mutations of EGFRs reported in up to 30% of solid tumors (including breast, colorectal, lung, pancreatic, gastric, head and neck cancer, and glioblastomas) generally correlate with a poor prognosis [5,6]. EGFR mutations have been found in the tyrosine kinase domain of EGFRs. Almost all patients who initially respond to EGFR-tyrosine kinase inhibitors (EGFR-TKIs) develop resistance to these drugs by acquiring EGFR mutations [7]. Resistance to the other anti-EGFR therapies can also occur through anti-stress mechanisms by cancer cells to overcome the cytotoxic effects of anti-EGFR therapies.

Autophagy is a self-digesting cellular process that allows cells to sequester cytoplasmic contents, through the formation of double membrane vesicles (autophagosomes). Autophagy as a survival mechanism provides an alternative energy source and facilitates the disposal of unfolded proteins during metabolic stresses [8,9]. Allelic loss of Beclin1, a mediator of autophagy, has been reported in various cancers [10], suggesting a close relationship between autophagy and cancer. Protective autophagy promotes resistance to anti-cancer drugs [11]. Receptor tyrosine kinase inhibitors (RTKi) are known to induce protective autophagy for cell survival [12]. A number of anti-cancer compounds such as RTKi can induce protective autophagy and result in resistance to these RTKi [13]. Erlotinib, the first-generation EGFR-TKI, can induce autophagy in sensitive NSCLC cells by activating EGFR mutations. Chloroquine, an inhibitor of autophagy, can enhance the effect of erlotinib in NSCLC cells [14]. In B-Raf proto-oncogene serine/threonine-protein kinase (BRAF) mutant (V600E) melanoma cells, a combination of BRAF inhibitor (BRAFi) with MEK inhibitor can induce protective autophagy. Autophagy inhibition is known to suppress the tumor growth of BRAF-resistant xenografts [15]. Therefore, targeting autophagy may overcome resistance to anti-EGFR treatments.

EGFR signaling both suppresses and promotes the autophagic response. All EGFR downstream signaling pathways are involved in autophagy modulation. The PI3K/AKT1 axis downstream of EGFR can inhibit autophagy by activating mTOR, an inhibitor of autophagy [16]. EGFR-mediated RAS signaling is known to promote autophagy [17]. EGFR-tyrosine kinase inhibitors (TKIs) and neutralizing antibody (EGFR monoclonal antibodies) treatments can induce autophagy in multiple cancers, including glioblastoma, human vulvar squamous carcinoma, colorectal adenocarcinoma, and NSCLC cells [18,19]. Among other mechanisms by which many tumors with EGFR mutation gain resistance to EGFR-tyrosine kinase inhibitors (EGFR-TKIs), autophagy suppression through EGFR-mediated Beclin1 (BECN1) phosphorylation can result in the homodimerization of Beclin1 [20,21].

This review focuses on the relationship between EGFR signaling and autophagy. We review recent reports concerning the emergence of autophagy as a mechanism of resistance to anti-EGFR treatments. We discuss the relevance of targeting both EGFR signaling and autophagy as a potential strategy for overcoming resistance to anti-EGFR treatments. We also review recent developments of therapeutics, such as chemicals, peptides, and microRNAs (miRNAs), that may overcome resistance to anti-EGFR treatments.

## 2. EGFR Structure and Mutations

**Figure 1:**
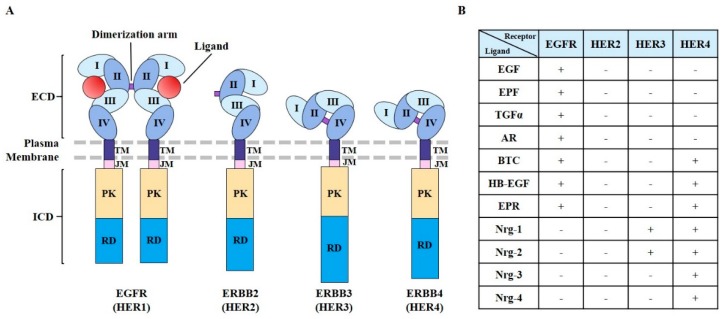
Structure of the human ErbB/HER receptors. (**A**) Extracellular domain (ECD) of each receptor consists of four domains (I–IV). Domains I and III participate in ligand binding (except for those of HER2). Domain II participates in dimer formation. Intracellular domain (ICD) is composed of protein kinase domain (PKD) and regulatory domain (RD). HER3 does not have active kinase domain. (**B**) Growth factors that can bind to the ErbB/HER receptors are indicated. AR, amphiregulin; BTC, betacellulin; EGF, epidermal growth factor; EPF, extracellular protein factor; EPR, epiregulin; HB-EGF, heparin-binding epidermal growth factor-like growth factor; Nrg-1/2/3/4, neuregulin-1/2/3/4; TGF-α, transforming growth factor α. TM denotes transmembrane and JM denotes juxtamembrane segments.

EGFR plays critical roles in cell proliferation [22], differentiation [23], motility [24], and the development of vasculature [25]. EGFR is expressed in the plasma membrane. EGFR has also been found in the nucleus, endosomes, and mitochondria. It may exert different functions in these different subcellular localizations [26,27,28,29]. The human EGFR family consists of four members (HER1–4) that belong to the ErbB lineage of proteins [30,31]. These receptors display similar molecular structures (Figure 1A). They all have an extracellular, cysteine-rich ligand-binding domain, a single α-helix transmembrane domain, a cytoplasmic tyrosine kinase (TK) domain (in all receptors except HER3), and a carboxy-terminal signaling domain.

The extracellular ligand-binding domain of EGFR contains four distinct subdomains (I–IV) [32] (Figure 1A). Domains I and III participate in ligand binding. Domains II and IV participate in disulfide bond formation [33,34]. Domain II participates in homo- and heterodimer formation with ErbB family members [35]. In response to ligand binding, homodimerization and/or heterodimerization with other family members can activate the tyrosine kinase activity of EGFR [35]. Ligands for EGFR include EGF, extracellular protein factor (EPF), TGF-α, amphiregulin (AR), betacellulin (BTC), epiregulin (EPR), and heparin-binding EGF-like growth factor [36] (Figure 1B). HER2 does not have ligands (Figure 1B). Nrg-1 and Nrg-2 are ligands for HER3 [37] (Figure 1B).

**Figure 2:**
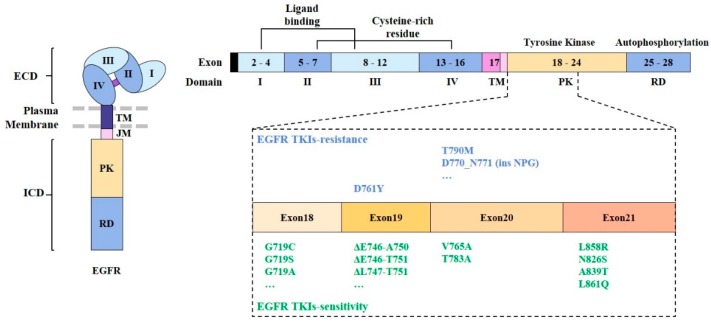
Domains of EGFR and the sites of mutations. Mutations that are associated with sensitivity or resistance to EGFR-TKIs are denoted. Specific mutations in the kinase domain of EGFR are shown. TKI denotes tyrosine kinase inhibitor.

EGFR mutations account for 10%–17% of NSCLC cases in North America and Europe and 30%–50% of NSCLCs in Asian countries. They are most common among patients with adenocarcinoma NSCLC and a light or non-smoking history [38,39]. Two independent studies [40,41] first reported the existence of somatic mutations in the tyrosine kinase (TK) domain of EGFR. These mutations are characterized by short deletions in exon 19 and point mutations (G719S, L858R, and L861Q) in exons 18 and 21 (Figure 2). Exon 19 class I deletions and exon 21 L858R mutations account for 85–90% of TK domain mutations [42]. In its inactive form, the EGFR kinase domain assumes a structure that results in the auto-inhibition of its activity [43]. Mutation at the TK domain of EGFR results in constitutive activation of its kinase activity, and activation of its downstream signaling pathways [44,45].

Activating mutations in the tyrosine kinase domain of the EGFR among NSCLC cells can enhance responses to EGFR-tyrosine kinase inhibitors (EGFR-TKIs) [46] (Figure 2). Point mutations in exon 18 [47,48], exon 20 [49], and exon 21 [50] are associated with sensitivity to EGFR-TKIs (Figure 2). Short deletions in exon 19 also confer sensitivity to EGFR-TKIs [51].

EGFR D761Y mutation is associated with resistance to first-generation EGFR-TKIs such as gefitinib and erlotinib [52] (Figure 2). EGFR G796D mutation [53] and short insertion in exon 20 are associated with resistance to erlotinib and gefitinib [48]. EGFR T790M mutation, a secondary acquired mutation, can result in resistance to gefitinib treatment [54]. EGFR T790M mutation is associated with resistance to first- and second-generation EGFR-TKIs. Cetuximab, an anti-EGFR mAb, can overcome resistance to these EGFR-TKIs [55].

There have been considerable advances in the treatment of NSCLC patients with EGFR mutations. Acquired resistance to EGFR-TKIs such as gefitinib and erlotinib is a critical obstacle in the treatment of EGFR mutant-positive NSCLCs.

## 3. EGFR Signaling

**Figure 3:**
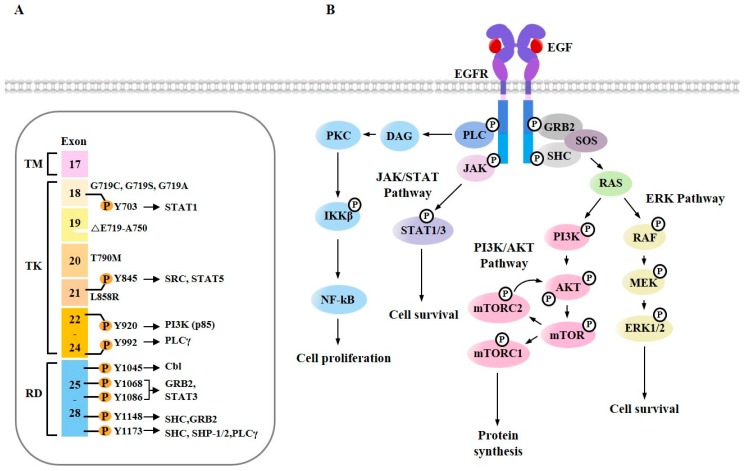
Ligand-dependent EGFR signaling pathways. (**A**) The proteins recruited on tyrosine-phosphorylated EGFR residues are shown. Numbers correspond to amino acids of EGFR. TM denotes transmembrane domain, TK denotes tyrosine kinase domain, and RD denotes regulatory domain. Specific mutations in the kinase domain and regulatory domain of EGFR are shown. Δ denotes deletions. (**B**) EGFR activates RAS/MAPK and JAK/STAT signaling pathways for cell survival. Activation of PI3K/AKT/mTOR signaling pathway leads to protein synthesis. EGFR activates Phospholipase C gamma (PLCγ), which in turn activates PKC/IKKβ/NF-κB signaling pathway.

Signaling from EGF plays critical roles in growth, survival, proliferation and differentiation [56], anti-cancer drug resistance [57], tumorigenesis [58], and metastasis [59]. EGF triggers EGFR dimerization and the phosphorylation of multiple tyrosine residues in its cytoplasmic tail (Figure 3A). Phosphorylated tyrosine residues can serve as docking sites for various cytoplasmic proteins, such as GRB2 (Growth factor receptor binding protein 2), SOS (Son of Sevenless), SHC (Src homology 2-containing), PLC (Phospholipase C), and JAK (Janus kinase) (Figure 3A). EGFR signaling triggers RAS/RAF proto-oncogene serine/threonine-protein kinase (RAF)/MEK/MAPK, JAK/signal transducer and activator of transcription (STAT), Protein Kinase C (PKC), and PI3K/AKT/mTOR (Figure 3B). RAS activated by EGFR can bind to RAF. This interaction activates mitogen-activated protein kinase kinase 1 (MEK1) and extracellular signal-regulated kinases 1/2 (ERK1/2) [60] (Figure 3B). PLCγ1 binds to EGFR through its Src Homology 2 (SH2) domain, hydrolyzes phosphatidyl inositol 4, 5-bisphosphate (PIP2) to diacylglycerol (DAG) and inositol 1, 4, 5-triphosphate (IP3). DAG activates PKC (Figure 3B). PKC increases the phosphorylation of inhibitor of nuclear factor kappa-B kinase subunit beta (IKKβ), which then leads to the activation of nuclear factor kappa-light-chain-enhancer of activated B cells (NF-κB) (Figure 3). The inhibition of NF-κB confers sensitivity to erlotinib [61]. This indicates that EGFR-TKIs target EGFR signaling. An activated AKT targets the mammalian target of rapamycin (mTOR) (Figure 3B). The inhibition of mTOR signaling induces autophagic apoptosis [62].

## 4. Cross Talk between EGFR Signaling and Autophagy

Autophagy is a conserved catabolic process that involves self-digestion and the removal of dysfunctional proteins and organelles [63]. Hypoxia [64], reactive oxygen species [65], and unfolded protein response [66] can induce autophagy to eliminate harmful stimuli. Reactive oxygen species may be due to electrons from dysfunctional mitochondria. Autophagy can eliminate reactive oxygen species [67,68]. Autophagy deficiency leads to the accumulation of dysfunctional mitochondria [69]. Reactive oxygen species (ROS) can induce autophagy and this leads to both non-apoptotic cell death and cell survival [70]. Autophagy might be a mechanism of cell survival [71]. Three types of autophagy have been reported: macroautophagy, microautophagy, and chaperone-mediated autophagy (CMA) [72]. A chaperone protein can bind to the cytosolic target substrate of CMA. It then binds to a receptor on the lysosomal membrane where the unfolding of the protein takes place. Next, the unfolded cytosolic target protein is delivered directly into the lysosome for degradation [73]. Microautophagy involves transferring cytosolic components into the vacuole or lysosome for degradation by either the direct protrusion/septation and/or invagination of the lysosomal or vacuolar membrane [74].

**Figure 4:**
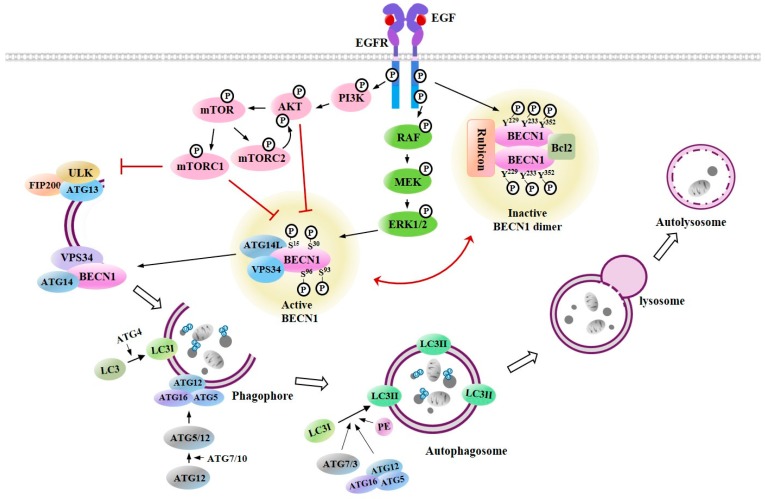
Cross talk between EGFR signaling and autophagy. Tyrosine phosphorylation of Beclin1 by EGFR leads to homodimerization of Beclin1 and binding of inhibitors of autophagy such as rubicon and B-cell lymphoma 2 (Bcl-2) to Beclin1 to decrease autophagic activity. EGFR-PI3K/AKT/mTOR signaling can inhibit autophagy by inhibiting phosphorylation of Beclin1 on serine residues. EGFR-RAF/MEK/extracellular signal-regulated kinases (ERK) signaling can activate autophagy by increasing serine phosphorylation of Beclin1. Beclin1-containing class III PI3 kinase complex initiates formation of autophagosomes. Autophagophore formation requires ATG12-ATG5-ATG16L1 conjugate. ATG4 cleaves microtubule-associated protein 1-light chain 3 (LC3) at the C-terminus to result in formation of LC3I, which is conjugated with phosphatidyl ethanolamine (PE) to become LC3II. LC3II is present on autophagosomes. Autophagosome fuses with lysosome to form autolysosome where intracellular contents are degraded and are recycled.

During macroautophagy, cytoplasmic components are bordered by phagophores, which enlarge and seal to form autophagosomes (Figure 4). These autophagosomes can fuse with lysosomes to form autolysosomes in which cytoplasmic components are degraded by resident hydrolases. Autophagy-related genes (ATGs) can facilitate the regulation of autophagy machinery [75]. The formation of autophagosome is initiated by unc-51-like kinase (ULK) and class III PI3-kinase (VPS34) complexes (Figure 4). The ULK complex is formed from ATG13, ATG101, ULK1/2, and family-interacting protein FIP200 [76] (Figure 4). ULK leads to the phosphorylation of Beclin1 and activates the PI3K (VPS34) complex [77] (Figure 4). Mammalian target of rapamycin complex 1 (MTORC1) can block the formation of the ULK complex and inhibit autophagic activity [78] (Figure 4). Small molecule activators of ULK can promote protective autophagy [79]. Autophagophore elongation and conversion into autophagosome requires ubiquitin-like conjugates. ATG12 can form a conjugate with ATG5 under the control of ATG7 and ATG10 (Figure 4). The resulting ATG12-ATG5 complex can interact with ATG16L1, forming a multimeric ATG12-ATG5-ATG16L1 conjugate that is on the outer surface of the autophagosomal membrane (Figure 4). Microtubule-associated protein 1-light chain 3-I (LC3-I) is conjugated with phosphatidyl ethanolamine (PE) by ATG7 and ATG3 to form membrane-bound LC3-II (Figure 4).

EGFR tyrosine kinase can induce the tyrosine phosphorylation of Beclin1 (pY229, pY233, and pY352), forming Beclin1 homodimers, and inhibiting autophagy (Figure 4). The tyrosine phosphorylation of Beclin1 triggers the disassembly of the Beclin1-VPS34 complex and leads to the inhibition of autophagy (Figure 4). Rubicon and Bcl-2 then bind to Beclin1 and act as negative regulators of autophagy [80] (Figure 4). EGFR activates the mechanistic target of the rapamycin complex 1 (mTORC1) pathway (Figure 4) and can act as a negative regulator of autophagy [81,82,83]. The active mTORC1 complex will decrease Beclin1-VPS34 activity, inactivate ULK1, and suppress autophagy [84] (Figure 4). MTORC2 leads to the stabilization and activation of AKT1 [85] (Figure 4). EGFR/RAS/RAF/MEK/ERK signaling activates autophagy by inducing the serine phosphorylation of Beclin1 (Figure 4). These findings suggest that there is a cross talk between EGFR signaling and autophagy.

## 5. Anti-Cancer Drugs Targeting EGFR and EGFR Signaling

**Figure 5:**
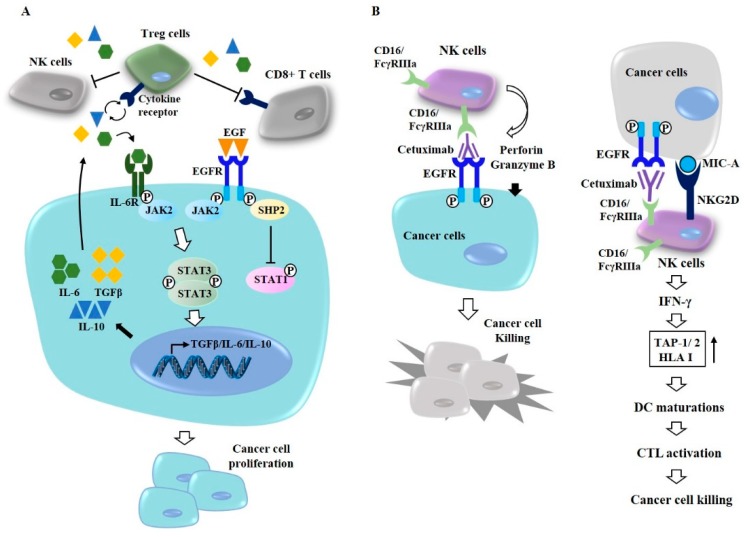
Antibody-dependent cell-mediated cytotoxicity mediates cytotoxic effect of cetuximab. (**A**) Upon binding of EGF, EGFR activates JAK2, which in turn leads to the activation of STAT3. STAT3 increases expression of immune suppressive cytokines, such as IL-6, IL-10, and TGF-β. IL-6, in autocrine fashion, binds to and activates IL-6 receptor. This binding activates STAT3. EGFR signaling activates regulatory T cells (Treg cells). Activated Treg cells suppress cytotoxic effects of natural killer cells (NK cells) and CD8+ T cells. IL-10 and TGF-β can also activate Treg cells. (**B**) Cetuximab-activated NK cells display cytotoxic effects against cancer cells by perforin and granzyme B (left). Cetuximab-activated NK cells interact with cancer cells through natural killer group 2 member D (NKG2D)- MHC class I chain-related gene A (MIC-A) binding. This interaction induces dendritic cell (DC) maturation by increasing expression levels of transporter 1 ATP-binding cassette sub-family B(TAP-1)/transporter 2 ATP-binding cassette sub-family B (TAP-2) and human leukocyte antigen I (HLA I) via interferon (IFN)-γ. DC maturation enhances cytolytic T lymphocytic activity towards cancer cells (right).

At present, EGFR-targeting anti-cancer drugs consist of two types: (1) EGFR monoclonal antibodies that inhibit the activation of the EGFR ligand-binding domain [86]; and (2) small-molecule EGFR-TKIs that inhibit the tyrosine kinase activity of the EGFR intracellular domain. EGFR-mediated signal transducer and activator of transcription 3 (STAT3) activation induces the expression of transforming growth factor-β (TGF-β), interleukin 6 (IL-6) [87], and interleukin 10 [88] in many tumors. IL-6, IL-10, and TGF-β levels increased by the EGFR-JAK2-STAT3 signaling pathway can activate regulatory T (Treg) cells, which in turn can inhibit the activity of cytotoxic T cells (CD8+ T cells) (Figure 5A). Src homology region 2 domain-containing phosphatase-2 (SHP2) activated by EGFR can inhibit the activation of STAT1 (Figure 5A). Inhibition of CD8+ T cells by Treg cells may confer protection against the cytotoxic effects of cetuximab (Figure 5A).

Cetuximab, a murine–human chimeric antibody, can bind to the extracellular domain of EGF receptor [89] (Figure 5B). Combined inhibition of EGFR and JNK/MAPK can overcome resistance to cetuximab [90]. Natural killer (NK) cells can interact with cancer cells through FcγRIIIa (CD16) which binds to cetuximab (Figure 5B). Cetuximab-activated NK cells display a cytotoxic effect toward cancer cells by perforin and granzyme (Figure 5B, left). NK cells activated by cetuximab display cytotoxic effects toward NSCLC and osteosarcoma [91,92]. Imgatuzumab, a novel glycoengineered anti-EGFR antibody, can induce antibody-dependent cell-mediated cytotoxicity (ADCC) and inhibit the growth of NSCLC cells [91]. Cetuximab-activated NK cells through natural killer group 2 member D (NKG2D)/MHC class I chain-related gene A (MICA) binding can also increase the expression levels of transporter 1 ATP-binding cassette sub-family B (TAP-1)/transporter 2 ATP-binding cassette sub-family B (TAP-2), induce dendritic cell (DC) maturation, and enhance CD8+ cytotoxic T lymphocyte (CTL) activity in an interferon (IFN)-γ-dependent manner [93] (Figure 5B, right). Panitumumab, a fully human Immunoglobulin G2a (IgG2a) mAb, also prevents ligands from binding to EGFR [94]. Unlike cetuximab, panitumumab does not induce ADCC. Zalutumumab, an IgG1 anti-EGFR mAb, can inhibit ligand binding, EGFR signaling, and the metastatic potential of lung cancer cells [95]. Although these mAbs have shown anti-cancer effects, these anti-EGFR mAbs need further clinical validations.

**Figure 6:**
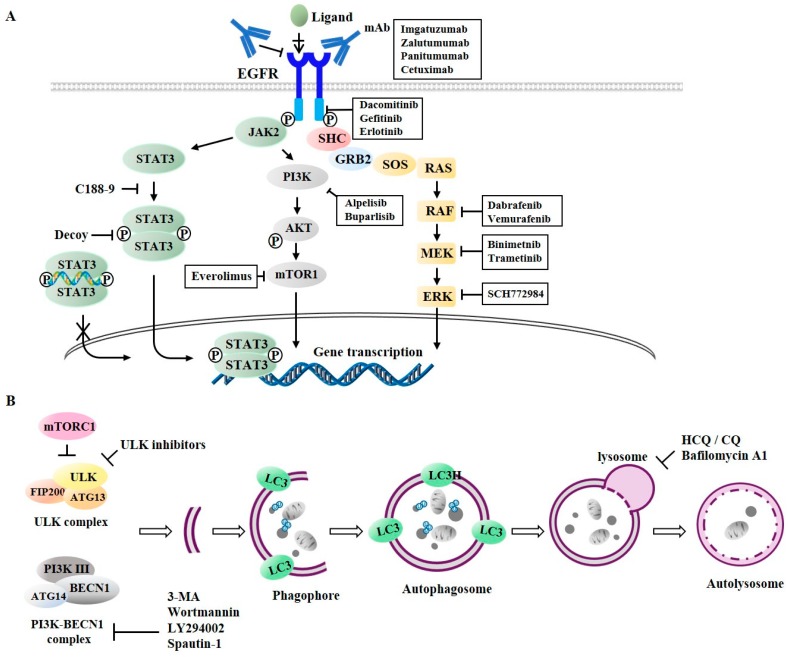
Anti-cancer drugs targeting EGFR signaling and autophagy. (**A**) Anti-EGFR monoclonal antibodies (mAbs) targeting EGFR include cetuximab, panitumumab, zalutumumab, and imgatuzumab. These mAbs bind to extracellular domain of EGFR and inhibit binding of ligands to EGFR. EGFR-TKI such as erlotinib targets intracellular tyrosine kinase domain to inhibit autophosphorylation of EGFR. Buparlisib and Alpelisib target PI3 Kinase. Everolimus targets mTOR activity and PI3K signaling. SCH772984 targets ERK activity. (**B**) Wortmannin, 3-methyladenine (3-MA), and LY294002 inhibit VPS34 complex formation. Spautin-1 promotes its degradation. Hydroxychloroquine (HQ), chloroquine (CQ), and bafilomycin A1 inhibit fusion of autophagosome with lysosome.

Dacomitinib (Figure 6A), a second generation irreversible inhibitor of EGFR-1, -2, and -4 tyrosine kinase, was approved for the treatment of NSCLC patients with EGFR mutations (EGFR exon 19 deletion or exon 21 L858R substitution mutations) [96]. Dabrafenib and vemurafenib as inhibitors of RAF (Figure 6A) can enhance the anti-cancer effects of second-generation EGFR-TKIs (lapatinib and afatinib) in colorectal cancer cells [97]. Inhibition of EGFR-MEK signaling pathway leads to the downregulation of Bcl-2 and induced myeloid leukemia cell differentiation protein Mcl-1 (Mcl-1) and promotes apoptosis to confer sensitivity to anti-EGFR treatments [98]. Binimetinib, an inhibitor of MEK1/2 (Figure 6A), can enhance the effect of cetuximab in neuroblastoma RAS viral oncogene homolog (NRAS)-mutant colorectal cancer cells [99]. Trametinib (an inhibitor of MEK1/2) in combination with erlotinib can suppress the invasion potential of intestinal adenocarcinoma [100]. PD-L1 (immune check point) expression is increased in erlotinib-resistant NSCLC cells. Such increased expression of PD-L1 is reversed by SCH772984, an inhibitor of ERK [101]. It is known that RAF and MEK co-inhibition exhibits synergy in triple negative breast cancer (TNBC) models [102].

STAT3 mediates EGFR signaling (Figure 6A). Inhibition of EGFR/STAT3 activation overcomes resistance to EGFR-TKIs in various NSCLC cells [103]. C188-9 can inhibit the dimerization of STAT3 [104] and decrease the in vivo tumorigenic potential of NSCLC cells [105]. A STAT3 decoy consisting of double stranded oligonucleotides can bind to STAT3 and prevent STAT3 from binding to target gene promoters [106,107]. Buparlisib (an inhibitor of PI3K signaling) in combination with erlotinib exerts anti-tumor effects [108]. Alpelisib, an isoform-specific PI3K inhibitor (amti-p110α), is in clinical trials [109]. Everolimus, an inhibitor of mTOR, can inhibit AKT signaling. In combination with cetuximab, it can suppress tumor growth [110]. Everolimus reverses resistance to erlotinib in lung adenocarcinoma cells [111]. Table 1 presents several examples of anti-cancer drugs, their targets, and indications. These reports indicate that targeting EGFR signaling may enhance the anti-proliferative effects of anti-EGFR treatments.

## 6. Autophagy and Anti-Cancer Drug Resistance

Resistance to erlotinib and gefitinib occurs through EGFR T790M mutation. This mechanism (i.e., EGFR T790M mutation) accounts for approximately 50% of the resistance to these EGFR-TKIs in NSCLC cells [112]. EGFR T790M mutation does not affect the downstream signaling of EGFR. The inhibition of the downstream signaling of EGFR does not enhance sensitivity to these EGFR-TKIs [113]. Therefore, other mechanisms are associated with resistance to erlotinib and gefitinib by EGFR T790M mutation.

Autophagy may provide cells with energy to survive a stress condition (such as under therapy with anti-cancer drugs) by recycling their own proteins and damaged organelles. Increased expression of Sequestosome 1 (SQSTM1/p62), a selective adaptor protein in autophagy, is associated with a poor response to cetuximab therapy [114]. Increased autophagic flux is associated with resistance to anti-cancer therapies, including radiation therapy and chemotherapy [115,116,117]. Cancer cells may induce autophagy as a survival mechanism in response to anti-cancer therapies [118,119,120]. Protective autophagy confers resistance to erlotinib in head and neck squamous cell carcinomas [121]. It also confers resistance to AG1478, an inhibitor of EGFR tyrosine kinase, in ovarian cancer cells [122]. Microtubule associated protein 1 light chain 3 alpha (LC3A)-mediated autophagy in carcinoma cells confers resistance to EGFR-TKIs [123]. Anti-EGFR treatments can induce protective autophagy in several cancer cell lines and solid tumors [124]. Osimertinib, a covalent and an irreversible EGFR-TKI, is a third-generation EGFR-TKI that has been approved for the treatment of NSCLC patients with EGFR T790M, L858R, and exon 19 deletion mutations. Enhanced autophagy is associated with resistance to osimertinib both in vitro and in vivo [125]. Osimertinib can induce an autophagy and decrease apoptosis by generating ROS in NSCLC cells [126]. These reports suggest that targeting autophagy may confer sensitivity to anti-EGFR treatments.

## 7. Targeting Autophagy Overcomes Resistance to Anti-EGFR Treatments

Tumors treated with single agents usually develop resistance. In lung cancer cells, autophagy may be induced by EGFR-TKIs. Co-inhibition of EGFR signaling and autophagy has demonstrated promising results in vitro [27,127]. Autophagy inhibitors can prevent autophagosome formation or block autophagosome–lysosome fusion (Figure 6B). Wortmannin, 3-methyladenine (3-MA), and LY294002 can inhibit the formation of VPS34 complex [128] (Figure 6B), whereas spautin-1 promotes its degradation [129]. The small-molecule inhibitor of ULK1 can prevent tumor metastasis and confers sensitivity to various anti-cancer drugs in neuroblastoma cells [130]. The inhibition of AKT by wortmannin, an inhibitor of autophagy, can decrease the expression of Rad51 and enhance the sensitivity of erlotinib-resistant NSCLC cells to erlotinib [131]. LY294002 can enhance sensitivity to salinomycin by decreasing AKT activity and suppressing autophagy [132]. Salinomycin is known to be able to enhance sensitivity of NSCLC cells to erlotinib by inhibiting AKT activity [133]. Autophagy inhibition by spautin-1 enhances sensitivity to oncogenic receptor tyrosine kinase inhibitors (RTKis) in neuroblastoma cells [134].

Hydroxychloroquine (HCQ), chloroquine (CQ), and bafilomycin A1 can inhibit the fusion of autophagosome with lysosome [128] (Figure 6B). Lapatinib and gefitinib can induce protective autophagy. In combination with autophagic inhibitors (CQ, 3-MA, or bafilomycin A1), lapatinib and gefitinib can enhance the anti-cancer effects of these EGFR-TKIs in bladder cancer cells [135]. Autophagy inhibition using chloroquine (CQ) and 3-MA can enhance the cytotoxicity of afatinib, a second-generation tyrosine kinase inhibitor (TKI), suggesting a protective role of autophagy in lung adenocarcinoma therapy with afatinib [136]. Autophagy inhibition by chloroquine (CQ) can enhance the sensitivity of NSCLC cells to erlotinib [137] and gefitinib [138]. Inhibition of autophagy by CQ and 3-MA can enhance the sensitivity of glioblastoma cells to erlotinib [139]. SB02024, a novel selective inhibitor of VPS34, can enhance the cytotoxic effect of erlotinib on breast cancer cells [140]. Bafilomycin A1, an inhibitor of vacuolar adenylpyrophosphatase (vATPase), can enhance the effects of EGFR-TKIs on NSCLC cells [141]. Cepharanthine, a novel autophagy inhibitor, can enhance the sensitivity of NSCLC cells to EGFR-TKI dacomitinib by blocking autophagosome–lysosome fusion [142]. The inhibition of cetuximab-induced autophagy by downregulating autophagy-related genes (ATG) or treating cancer cells with lysosomal inhibitors can enhance cetuximab-induced apoptosis [143]. Table 2 presents several examples of anti-autophagic drugs, their targets, indications, and drugs exerting synergistic effects with anti-autophagic drugs. These reports suggest that autophagy inhibition may enhance the sensitivity of various cancer cells with EGFR mutations to anti-EGFR treatments.

## 8. MiRNAs Regulate Response of Cancer Cells to Anti-EGFR Treatments

MiRNAs (micro RNAs), 21–23 nucleotides in length, are endogenous non-coding RNAs that can regulate the expression of target genes either through the translational inhibition or destabilization of messenger RNA (mRNA) [144]. MiRNAs can regulate tumorigenesis and cellular proliferation [145]. Many reports have suggested the roles of miRNAs in anti-cancer drug resistance and autophagy. MiR-375 can enhance the effect of cetuximab on the proliferation of colorectal cancer cells [146]. MiR-43 or miR-145 can enhance the sensitivity of colon cancer cells to cetuximab by enhancing ADCC induced by cetuximab [147]. MiR-199a-5p and miR-375 can confer resistance to cetuximab by targeting PH domain and leucine-rich repeat protein phosphatase 1 (PHLPP1) in colon cancer cells [148]. MiR-200c can overcome resistance to trastuzumab in breast cancer cells [149]. MiR-7 targets EGFR and enhances the effect of cetuximab on colorectal cancer cells [150]. MiR-204 blocks JAK signaling and enhances the sensitivity of head and neck cancer cells to cetuximab [151].

Tumor suppressor miRNAs, such as let-7 and miR-34, can enhance the anti-cancer effects of erlotinib on NSCLC cells [152]. MiR-217 can enhance the sensitivity of melanoma cells to gefitinib and trastuzumab by inhibiting the interaction of EGFR with cancer/testis antigen CAGE [153]. MiR-495-3p targets mTOR signaling and decreases multidrug-resistance phenotypes [154]. MiR-199a targets ephrin type-A receptor 2 (EphA2) and enhances sensitivity to erlotinib [155]. MiR-9 exerts inhibitory effects on erlotinib [156]. MiR-30a enhances sensitivity to gefitinib and erlotinib by regulating PI3K/AKT pathway and inhibits the migration and invasion potential of cancer cells [157]. MiR-143-3p and miR-373-5p can enhance the sensitivity of erlotinib-resistant non-small cell lung carcinoma cells (PC-9/ER) cells to erlotinib and osimertinib [158]. MiR-608 and miR-4513 can enhance the sensitivity of NSCLC cells, such as PC-9 cells, to gefitinib [159]. MiR-873 confers sensitivity to gefitinib in NSCLC cells by targeting glioma-associated oncogene homolog 1 [160]. MiR-483-3p can enhance the sensitivity of NSCLC cells to gefitinib by increasing autophagy and apoptosis [161]. MiR-123 inhibitor is known to increase the expression of phosphatase and tensin homolog (PTEN, a negative regulator of PI3K/AKT signaling) and enhance the sensitivity of lung cancer stem cells to erlotinib [162]. MiR34a acts as a tumor suppressor. The combination of miR-34a mimic and EGFR-TKIs (such as afatinib, rociletinib, and osimertinib) can enhance the sensitivity of NSCLC cells to these EGFR-TKIs [163]. Since miRNAs can regulate EGFR signaling and/or autophagy, they can be developed as anti-cancer drugs for overcoming resistance to anti-EGFR treatments.

MiRNAs that show differential expression between anti-cancer drug-sensitive and anti-cancer drug-resistant NSCLC cells have been identified [164]. These miRNAs may affect autophagy and regulate the responses of various cancers to anti-EGFR treatments. MiRNAs that can predict the response of head and neck cancer to panitumumab have been identified in a clinical trial [165]. These miRNAs can be employed as drugs for overcoming resistance to anti-EGFR treatments. It would be also necessary to identify molecules regulated by anti-EGFR treatments by employing RNA sequencing, miRNA arrays, and cytokine arrays. These molecules may affect autophagy to regulate the responses of various cancers to anti-EGFR treatments.

Exosomes are extracellular vesicles (EVs) produced in the endosomal compartment of eukaryotic cells. Multivesicular body (MVB) is an endosome that buds inward into the endosomal lumen. If MVB fuses with the plasma membrane, these vesicles are released as exosomes. Recent reports suggest a role of inactive EGFR in autophagy, which is stimulated by serum starvation and EGFR-TKIs [166]. Endosomal EGFR accumulation is made possible by an interaction between EGFR and lysosomal-associated protein transmembrane 4 beta (LAPTM4B). Endosomal EGFR can interact with rubicon (an inhibitor of autophagy) and induce the disassociation of rubicon from Beclin1, leading to the activation of Beclin1 and the initiation of autophagy [166]. These reports suggest that exosomes play important roles in autophagy and anti-cancer drug resistance. Cancer cells can release exosomes containing unique molecular features, such as miRNAs and cytokines. Cetuximab can decrease the expression levels of EGFR and phospho-EGFR (pEGFR) in the exosomes [167]. Treatments of cancer cells with second-generation EGFR-TKIs, such as CI-1033 and PF-00299804, can induce the secretion of exosomes containing EGFR, pEGFR, and exosomal DNAs [168]. These molecules may transfer EGFR-TKI resistance to sensitive cancer cells. Erlotinib resistance can be transferred via exosomes containing long non-coding RNA (lncRNA) RP11 838N2.4 [169]. Exosomes of gefitinib-treated NSCLC cells can inhibit the anti-tumor effect of cisplatin [170]. The identification and functional characterizations of exosomal miRNAs/cytokines in cancer cells treated with anti-EGFR treatments may offer a valuable strategy for overcoming resistance to anti-EGFR treatments.

## 9. Anti-Autophagic Peptides That Regulate Response to Anti-EGFR Treatments

CAGE, a cancer/testis antigen, can confer resistance to various anti-cancer drugs in melanoma cells. CAGE binds to EGFR and HER2. The downregulation of EGFR enhances the sensitivity of melanoma cells to anti-cancer drugs [153]. CAGE binds to EGFR, directly regulates the expression of EGFR, and confers resistance to anti-cancer drugs in melanoma cells [171]. CAGE confers anti-cancer drug resistance by promoting autophagic flux in breast cancer cells [172]. CAGE binds to Beclin1 and regulates responses to erlotinib in erlotinib-resistant NSCLC cells, such as PC-9/ER [158]. These reports indicate a role of CAGE in autophagy and suggest that CAGE can be a target to develop therapeutics for patients with cancer that is resistant to anti-EGFR treatments. CAGE-derived AQTGTGKT peptide (^266^AQTGTGKT^273^) corresponds to DEAD box domain of CAGE, binds to CAGE, inhibits the interaction between CAGE and Beclin1, decreases autophagic flux in PC-9/ER cells, and enhances the sensitivity of PC-9/ER cells to erlotinib and osimertinib [158]. CAGE-derived GTGKT peptide (^269^GTGKT^273^) corresponds to DEAD box domain of CAGE, can inhibit the interaction between CAGE and glycogen synthase kinase 3 beta (GSK3β) and enhance the sensitivity of anti-cancer drug-resistant melanoma cells to anti-cancer drugs [173]. AQTGTGKT and GTGKT peptides correspond to the domain of CAGE necessary for interaction with EGFR [173]. It will be interesting to further discover peptides that can bind to CAGE and inhibit the interaction of CAGE with EGFR, GSK3β, or Beclin1. Combination of anti-EGFR treatments with these peptides may enhance sensitivity to anti-EGFR treatments.

Distinct subgroup of the Ras family (DIRAS) can act as a tumor suppressor. DIRAS binds to Beclin1 and forms an autophagy initiation complex. DIRAS-derived peptides can bind to Beclin1 and inhibit DIRAS-mediated autophagy [174]. These DIRAS-derived peptides may confer sensitivity to anti-EGFR treatments. Peptides that can bind to Beclin1 or other molecules involved in autophagy may enhance the sensitivity of cancers to anti-EGFR treatments.

## 10. Conclusions and Perspectives

EGFR has emerged as a valuable target for the development of anti-cancer drugs. EGFR-targeting antibodies (such as cetuximab and panitumumab) and EGFR-TKIs (such as gefitinib, erlotinib, afatinib, and osimertinib) have been developed. First generation EGFR-TKIs have shown promising effects on cancer patients with EGFR L858R mutation [175]. However, resistance to these EGFR-TKIs can occur through acquired EGFR T790M mutation. The second-generation EGFR-TKIs (afatinib, neratinib, and dacomitinib) target EGFR T790M mutation and other EGFR activating mutations. Combination therapy of a new second-generation EGFR-TKI with cetuximab is highly effective against tumors bearing EGFR T790M mutation [176]. The clinical values of these EGFR-TKIs remain largely unknown.

**Figure 7:**
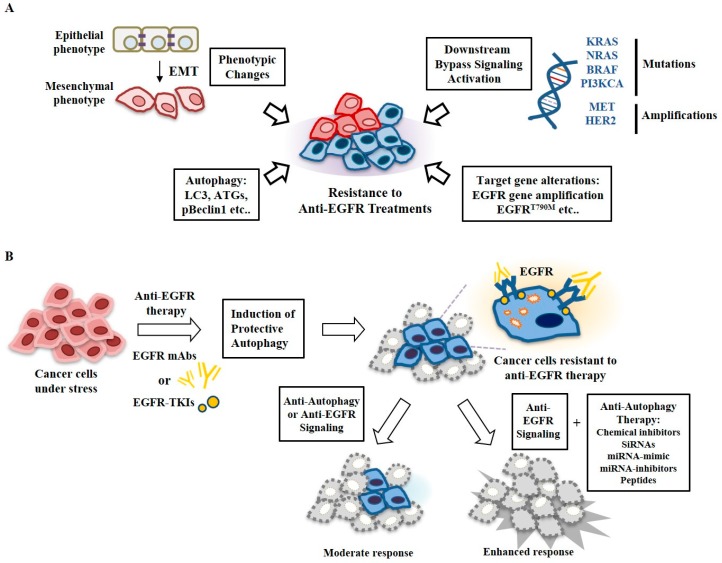
Targeting autophagy may overcome resistance to anti-EGFR treatments. (**A**) Resistance to anti-EGFR treatments develops from mutations and amplifications, phenotypic changes, and autophagy. (**B**) Anti-EGFR treatments such as anti-EGFR mAbs and EGFR-TKIs induce protective autophagy in cancer cells, which confers resistance to these anti-EGFR treatments. Targeting both EGFR and autophagy may overcome resistance to anti-EGFR treatments. Anti-autophagy therapy includes miRNA-mimic, miRNA-inhibitors, peptides, and small interfering RNAs (siRNAs).

Resistance to these EGFR-TKIs can also occur through downstream signaling activation of Kirsten rat sarcoma viral oncogene homolog (KRAS), NRAS, BRAF, phosphatidylinositol-4,5-bisphosphate 3-kinase catalytic subunit alpha (PIK3CA), and tyrosine-protein kinase Met (MET) mutations [177] (Figure 7A) and phenotypic changes, such as epithelial mesenchyme transitions [178] (Figure 7A). To develop drugs for overcoming resistance to anti-EGFR treatments, it will be necessary to determine molecular patterns, such as autophagic flux, EGFR signaling pathways, tumor suppressors, oncogenes, and mutations for each tumor. Autophagic flux can serve as a target to develop drugs for overcoming resistance to anti-EGFR treatments. Many reports have indicated that autophagy confers resistance to anti-EGFR treatments (Figure 7A). Small molecules that specifically target autophagic flux, such as ATGs, can overcome resistance to anti-EGFR treatments.

Understanding the functional association of autophagy with anti-EGFR resistance may provide a promising therapeutic strategy to enhance the effects of EGFR-TKIs in cancer patients with EGFR mutations and/or overexpression. Autophagy induced by anti-EGFR treatments can promote the survival of tumors with high levels of basal autophagy, ROS, and DNA damage or under metabolic stress (Figure 7A). Reports concerning cross talk between EGFR signaling and autophagy have been published. Recently, peptides and miRNAs that can regulate the response to anti-EGFR treatments and autophagic flux have been reported (Figure 7B). Peptides and miRNAs that target autophagy and EGFR signaling can be employed as therapeutics against patients with cancer that is resistant to anti-EGFR treatments. Combinations of anti-autophagic drugs with inhibitors of EGFR signaling can provide a novel treatment opportunity for cancers that are resistant to anti-EGFR treatments (Figure 7B) and these strategies should be clinically tested.

## Figures and Tables

**Table 1:** Summary of anti-cancer drugs targeting EGFR.

Drugs	Targets	Class	Indications	References
Imagatuzumab	EGFR: extracellular domain	mAb directed against EGFR	NSCLC	[91]
Zalutumumab			Lung cancers	[95]
Panitumumab			Head and Neck, Colorectal cancer	[94]
Cetuximab			NSCLC, Osteosarcoma	[55,89,90,91,92,93,99,110,111]
Dacomitinib	EGFR: tyrosine kinase domain	Small molecule	NSCLC	[96]
Gefitinib			NSCLC, Breast cancer	[48,52,54]
Afatinib			Head and Neck, Lung cancers	[97,111]
Erlotinib			Head and Neck, NSCLC	[14,48,52,61,100,101,108]
Dabrafenib	Inhibitor of EGFR signaling: RAF	Small molecule	Colorectal cancer	[97]
Vemurafenib			Colorectal cancer	[97]
Binimetnib	Inhibitor of EGFR signaling: MEK	Small molecule	Colorectal cancer	[99]
Trametinib			Intestinal adenocarcinoma	[100]
SCH772984	Inhibitor of EGFR signaling: ERK	Small molecule	NSCLC	[101]
Alpelisib	Inhibitor of EGFR signaling: PI3K	Small molecule	Breast, Rectal cancer	[109]
Buparlisib			Head and Neck	[108]
Everolimus	Inhibitor of mTOR	Small molecule	Colon cancer, lung cancer	[110,111]
C188-9	Inhibitor of STAT3	Small molecule	NSCLC, AML	[104,105]
Decoy		Oligonucleotide	Breast cancer	[106,107]

NSCLC, denotes non-small cell lung cancer; AML, denotes acute myeloid leukemia.

**Table 2:** Summary of anti-autophagic drugs.

Drugs	Targets	Mechanism of Action	Drug Combinations	Indications	References
3-MA	PI3K	Autophagosomes formation: PI3K-Beclin1 complex activity	Lapatinib, Afatinib, Erlotinib	Lung cancer, bladder cancer	[128,135,136,139]
Wortmannin			Erlotinib	NSCLC	[128,131]
LY294002			Salinomycin, Erlotinib	Prostate cancer	[128,132]
Spautin-1	USP10/13		RTKis (Afatinib, Sorafenib, TP-0903)	Neuroblastoma	[129,134]
SBI-0206965	ULK1		TRAIL	Neuroblastoma	[130]
HCQ/CQ	Lysosomes	Inhibition of autophagosome-lysosomes fusion	Erlotinib, Gefitinib	NSCLC, bladder cancer	[128,135,136,137,138,139]
Bafilomycin	Vacuolar ATPase		Erlotinib, Gefitinib, Lapatinib	NSCLC, bladder cancer	[128,135,141]
Cepharanthine	Lysosomes		Dacomitinib	NSCLC	[142]

ULK, unc-51-like kinase; USP, ubiquitin-specific protease.
